# Creating longitudinal datasets and cleaning existing data identifiers in a cystic fibrosis registry using a novel Bayesian probabilistic approach from astronomy

**DOI:** 10.1371/journal.pone.0199815

**Published:** 2018-07-09

**Authors:** Peter Donald Hurley, Seb Oliver, Anil Mehta

**Affiliations:** 1 Department of Physics and Astronomy, University of Sussex, Brighton, United Kingdom; 2 Division of Medical Sciences, University of Dundee, Dundee, United Kingdom; Cincinnati Children’s Hospital Medical Center, UNITED STATES

## Abstract

Patient registry data are commonly collected as annual snapshots that need to be amalgamated to understand the longitudinal progress of each patient. However, patient identifiers can either change or may not be available for legal reasons when longitudinal data are collated from patients living in different countries. Here, we apply astronomical statistical matching techniques to link individual patient records that can be used where identifiers are absent or to validate uncertain identifiers. We adopt a Bayesian model framework used for probabilistically linking records in astronomy. We adapt this and validate it across blinded, annually collected data. This is a high-quality (Danish) sub-set of data held in the European Cystic Fibrosis Society Patient Registry (ECFSPR). Our initial experiments achieved a precision of 0.990 at a recall value of 0.987. However, detailed investigation of the discrepancies uncovered typing errors in 27 of the identifiers in the original Danish sub-set. After fixing these errors to create a new gold standard our algorithm correctly linked individual records across years achieving a precision of 0.997 at a recall value of 0.987 without recourse to identifiers. Our Bayesian framework provides the probability of whether a pair of records belong to the same patient. Unlike other record linkage approaches, our algorithm can also use physical models, such as body mass index curves, as prior information for record linkage. We have shown our framework can create longitudinal samples where none existed *and* validate pre-existing patient identifiers. We have demonstrated that in this specific case this automated approach is *better* than the existing identifiers.

## Introduction

Registries are used to describe the clinical status of patients and foster care improvement [1] and are invaluable tools in the analysis of rare diseases because of the limited number of patients affected in a given geographical zone, as reviewed recently [2]. They are equally useful at the common end of the clinical spectrum, for example in Alzheimer’s disease, to aggregate sufficient numbers of patients into meaningful prognostic subgroups [3].

Longitudinal datasets are needed for disease modification studies and to better understand prognosis through the analysis of co-variance of different data variables. The current state of play with rare diseases is to amalgamate datasets from individual countries where data are usually collected annually, giving population ‘snapshots’. However, this only allows studies at the population level and does not permit the longitudinal studies that are required for outcome prediction. Constructing longitudinal samples remains a difficult challenge for registries given the retrospective nature of the underlying data sets, issues of data anonymity, and gaps or errors in the data through changed software, interrupted funding cycles, poor record keeping of legacy systems and inadequate staffing [4, 5]. There is thus a clear need to provide robust algorithms to facilitate prospective outcome analysis from cross sectional data.

In extragalactic astronomy, astronomers are interested in the properties of galaxies such as their position on the sky, how many stars they contain and the star formation rate, *SFR*. In order to answer these questions, astronomers take observations with telescopes that observe at different parts of the electromagnetic spectrum and measure how bright galaxies are at different wavelengths. Catalogues of galaxy position and brightness are made from each telescope and then cross matched such that for each galaxy, we know its position on the sky and how its brightness changes at different wavelengths. This allows us to fit models and constrain properties such as the *SFR*.

Typically, galaxy catalogues are matched based on their angular position on the sky (i.e. right ascension and declination). Positional cross matching different catalogues is not always straight forward. Over the last decade, space based telescopes such as the Spitzer Space telescope [6] and the ESA Herschel Space Observatory [7] have made observations at the far-infrared part of the electromagnetic spectrum. Because of the longer wavelength, and the limit to the size of telescope mirror for space-borne facilities, the resolution of these observations is low relative to those at lower wavelengths. This means the uncertainty on galaxy position becomes so large, matching on galaxy position alone is no longer appropriate.

**Fig 1:**
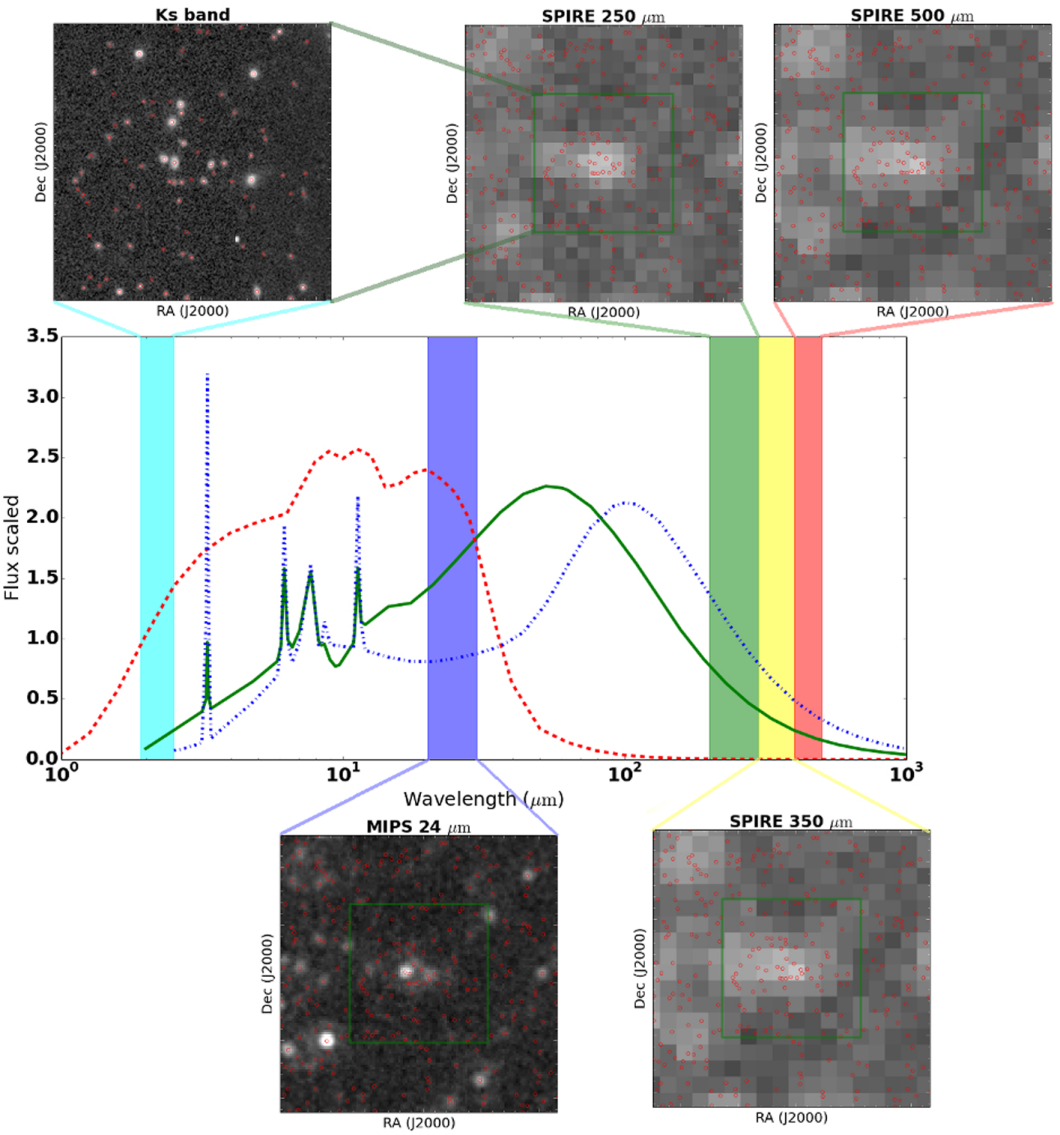
A galaxy catalogue based on the Ks band image from ULTRAVista (red circles), over plotted on the original Ks band image, a MIPS 24 micron image from Spitzer, and the three images taken with the SPIRE instrument on Herschel. The middle plot shows how flux varies as a function of wavelength, for three different types of galaxy models. We can use these models as prior information, to help us cross match sources from different telescopes.

This problem is demonstrated in Fig 1, where we show a galaxy catalogue (in red) on top of a near-infrared image (from ULTRAVista [8]), a mid infra red image (MIPS 24 micron image from Spitzer [9]), and the three far infrared images taken with the SPIRE [10] instrument on Herschel [11].

For the longer wavelength observations, it is difficult to ascertain which ‘blob’ belongs to which galaxy from position alone. To overcome this, we can make use of our ‘prior’ knowledge on how the brightness of different galaxies changes with wavelength. The middle plot in Fig 1 shows models for three different types of galaxies [12]. We can use these models to help us decide which galaxy is the best match to the ‘blob’ at longer wavelengths. This type of approach has been described in a formal Bayesian framework [13, 14], and we can adopt it to the problem of record linkage.

The analogy between the cross matching problem in astronomy and patient record linkage can be thought of as follows:

- Galaxies → Patients
- Wavelength → Time
- Redshift → Age
- Position → e.g. Gender, Genotype
- Brightness → e.g. Body Mass Index

This paper adapts the [13] framework and applies it to data contained within the European Cystic Fibrosis Society Patient Registry (ECFSPR) as a proof of concept. By using this framework, we show how physical models such as those that exist for Body mass index, can be used in the field of record linkage.

## Materials and methods

### Data

In order to validate our method, we compare it against a ‘gold standard’ dataset. The Danish subset of the ECFSPR was chosen for this study because of Denmark’s long tradition of high quality longitudinal registry data, in a setting of universal health care access. As such, the Danish registry is deemed as complete census [15]. The collection methods and format are described recently in [1]. Briefly, data are collected to a common proforma in two large Danish Centres caring for both children and adults with CF. The Danish database uses a non-anonymous unique identifier (ID) that cannot leave the country by law. An anonymized version of this data set is then transferred to the ECFSPR with manually created generic IDs in order to track patients across the years. We use these IDs as the original truth IDs to test our algorithm.

The data protection authorities gave permission for this anonymised analysis of the data by the ECFSPR. The Data Controller Dr Hanne Olesen granted permission for access to the Data after internal discussions with the the Danish Authorities and she should be contacted for further information via Registry Committee of the ECFS. The guidelines of the ECFS Patient Registry (ECFSPR) are made according to the Directive 95/46/EC of the European Parliament and of the Council of 24 October 1995 on the protection of individuals with regard to the processing of personal data and on the free movement of such data. The full guidelines and can be found on the ECFSPR site (https://www.ecfs.eu/projects/efcs-patient-registry/guidelines).

The dataset contained demographic data such as month and year of birth, sex, genotype of CF mutation along with annually collected clinical parameters such as lung function and body mass index. The registry does not contain person identifiable data such as name and address, but does internally hold details on what CF centre the patient attends for data quality and cleaning purposes. As with all projects using ECFSPR data, CF centre information is never made available to us due to anonymity reasons. We note however it could be included to improve accuracy if this record linkage were to be carried out internally to test data quality.

As with many disease registries, the data suffers from censoring situations such as patient moving country. We note death is recorded and is not an issue in terms of censoring. With respect to missing data, variables that do not change with year (e.g. phenotype and gender) are mostly complete across each year. Other variables that are time dependent such as BMI occasionally suffer from missing values.

We note that although the Danish dataset is the gold standard for the ECFSPR, there remain issues with the data quality when compared against the standards associated with typical electronic health records. The gold standard ECFSPR Danish dataset was created by data processors and clinical staff in Denmark. Like most rare disease registries, the ECFSPR is run by volunteers and local staff who may not be data experts. These data stretch back over decades and staff changes introduces variation in quality.

We use BMI, height, age at diagnosis and CFTR genotype (defined by the two alleles, with F508del/F508del representing about 50% of population across Europe) as variables in our cross matching, while assuming the date of birth variable in the ECFSPR, given to the month is correct. These variables have been chosen as they are the most complete in the database. With most CF sufferers now reaching adulthood, the bias that was previously inherent in BMI and height curves is now significantly reduced, allowing us to use them in our model [16].

### Record linkage

Strategies for record linkage can be subdivided into two: deterministic and probabilistic. The simplest methods are deterministic and require exact matching of variables. A good example of when a deterministic system is useful is during the matching of unique identifiers such as a social security number. If matching on several identifiers such as name, date of birth and postcode, then linkage scores are derived by predetermined rules. Deterministic methods require high quality data and do not take into account how likely values are to agree by chance [17]. Probabilistic record linkage methods are more advanced in that statistical properties are used to calculate the probability that the records apply to the same person and use linkage scores based on properties of variables being matched. The first probabilistic strategies for record linkage used the statistical framework introduced in [18]. Under this model, pairs of records are classified as links, possible links, or non-links using matching weights based on predetermined probabilities, however, algorithms based on this type of framework do not consider uncertainties [19], be it in input or output. More recently, Bayesian approaches to record linkage have shown to provide more robust handling and propagation of uncertainties [20, 21]. More recently, Bayesian hierarchical formalisms have been used to clean data of duplicates in database record linkage problems [22–24].

A Bayesian approach that utilises prior information, has been developed in Astronomy to solve the problem of cross matching galaxy catalogues by [13, 25]. Their general probabilistic formalism for cross-identifying astronomical point sources, allow the folding in of expert knowledge on the physics of objects in a hierarchical framework, to help in the cross-identification process. This was elaborated on by [14].

The idea of using prior information in the matching process is relatively new. For example, a Bayesian method for matching noisy multivariate normal vectors was illustrated in [26]. Our method takes a similar approach, but because of the specific case of matching in a disease registry where additional prior information is available, we can incorporate more sophisticated time varying models such as the previously constructed BMI and height curves.

Although developed within the field of astronomy, the framework introduced in [13] is general enough to be exploited in other fields that require cross-matching. Longitudinal registry databases are one such field. By using a mixture of cross sectional information contained within the databases, we hypothesised that we could use the particular framework developed in astronomy, to help cross match patients within registry databases across years without recourse to manual IDs. In the following section, we introduce our general Bayesian framework and how it applies to specific problems that occurs in rare disease registries. We use our framework to then provide a more thorough example of how the framework applies to discrete and continuous data found in the ECFSPR.

### Bayesian framework

In cross matching records across databases of different years, we are comparing all patient records in year one, against all patient records in year two. For each comparison of a pair of records, we test two hypotheses:

- *H*, the record from year 1 and record from year 2 are the same patient
- *K*, the record from year 1 and record from year 2 are not the same patient

In order to test the two hypotheses against each other, we use Bayesian probability rules.

### Bayesian probability

One of the fundamental relations in probability theory is Bayes’ rule. It provides the framework to update our belief in hypothesis *H*, given some relevant set of data variables, *D*, a model of how the variables are expected to behave, *M*, and any prior degree of belief in *H*:

$$\begin{array}{c} P\left(H|D,M\right)=\frac{p(D|M,H)\;P(H)}{p(D)} \end{array}$$ (1)

Where:

- *P*(*H*|*D*, *M*) is the posterior probability of *H*, given (denoted by |) a vector of data variables *D* and our model *M*
- *p*(*D*|*M*, *H*) is the likelihood of *D*, given *H* and *M*
- *P*(*H*) is the prior on *H*
- *p*(*D*) the prior probability of *D*, which normally is assumed as unknown and therefore a constant.

We can use this Bayesian framework to quantify whether hypothesis *H* or *K* is more believable. We do this by using the Bayes factor, *B*, defined as the ratio of the posterior over prior of *H* and *K*, which, after applying Bayes’ rule becomes:

$$\begin{array}{ccc} B(H,K|D,M) & = & \frac{p(D|H,M)}{p(D|K,M)} \end{array}$$ (2)

Eq 2 can be thought of as comparing the updated beliefs in *H* and *K* given our set of variables, *D*. To make Eq 2 more specific to our problem, we replace *D* with discrete variables such as gender and mutation, or continuous information such as BMI and height can use Eq 2 and use them to update our belief in *H* and *K*.

This Bayesian approach is inherently recursive. As soon as we obtain new measurements and compute the posterior probability, that becomes the prior for subsequent studies. This is an extremely powerful property and simplifies the computations enormously. A consequence of this is that the Bayes factor for conditionally independent variables can be combined by taking their product. When taking the logarithmic transformation of the Bayes factors, combining them becomes a summation.

As described in [13] and [27], if the hypothesis *H* and *K* are complementers of each other (i.e. *P*(*H*) + *P*(*K*) = 1 and *P*(*H*|*D*) + *P*(*K*|*D*) = 1), the Bayes factor naturally relates the prior probability, the comparison between the hypothesis *H* and *K* and posterior probability of our hypothesis via the relation:

$$\begin{array}{c} P(H|D,M)={\left[1+\frac{1-P\left(H\right)}{B(H,K|D,M)P\left(H\right)}\right]}^{-1} \end{array}$$ (3)

where *P*(*H*) is our prior. By calculating our combined Bayes factor and using an initial estimate for *P*(*H*), we calculate the posterior probability that one of our identifications is true.

We can estimate the prior probability by considering the process of picking the same patient from the two databases. If the number of patients that have true matches is *N*_*T*_ and the number of records in year one is *N*_1_ then the probability of picking a patient from year one which has a match in year two is *N*_*T*_/*N*_1_. If the number of records in year two is *N*_2_ then the probability of picking the same patient’s record from year two is 1/*N*_2_ (assuming there is no duplication of records). The combined probability of the two steps is *N*_*T*_/(*N*_1_ × *N*_2_). This is our prior. In reality, *N*_*T*_ is not known, however one can use self consistent examination by requiring that Σ*P*(*H*|*D*, *M*) = *N*_*T*_ and starting from an initial value of *N*_*T*_ = min(*N*_1_, *N*_2_). We then calculate *P*(*H*|*D*, *M*) using Eq 3 and repeat the procedure until we converge on a value for *N*_*T*_.

### Our complete recipe

At present, we calculate the Bayes factor for gender, BMI, height, genotype and age at diagnosis. Additional variables could be added as required. The date of birth variable in the ECFSPR is given to the month and we assume it is correct. This allows the cross matching to take place on a month by month basis, reducing the number of combinations. Thus, the algorithm for determining the final associations is as follows:

- For records with a date of birth in a specific month and if the data is present, we calculate the Bayes factor for gender, genotype, age at diagnosis and (if the same gender) height and BMI for every possible combination of records between the two years being matched.
- We add together ln *B*_*gender*_, ln *B*_*BMI*_, ln *B*_*height*_, ln *B*_*genotype*_ and ln *B*_*age*_*diag*_ to get the final Bayes factor ln *B*_*total*_ for each potential match.
- We convert our final Bayes factor to a probability using Eq 3 and carrying out the self consistency examination. Combinations with the highest probability and without duplication are taken as the final matches.

We note that in summing the log of Bayes factors for ECFSPR variables, we are treating them as conditionally independent. In reality this is unlikely to be true for all variables. For example, age at diagnosis could well be dependent on genotype. Other variables such as BMI and height can be thought as independent when not considering weight. They become conditionally dependent only if weight is introduced as an additional variable. In reality, dropping the independence assumption makes little difference but vastly increases the model complexity.

### Bayes factor calculation for gender

As an example for discrete data, we will go through the steps for calculating the Bayes factor for gender. A patient will usually remain the same gender across years. The only caveat to this is when patients change gender, or when records are incorrect, however we can use our full probabilistic framework to take this into account.

First, let us define our gender model, *M*. We can rewrite Eq 2 as:

$$\begin{array}{ccc} B(H,K,M|D) & = & \frac{p(D|M,H)}{p(D|M,K)} \end{array}$$ (4)

Our model, *M*, describes the probability of two records belonging to the same patient given the data, *D*, where *D* = {*g*_1_, *g*_2_} (the genders in the records from year 1 and year 2 respectively). Since there are only two outcomes, we can use the Bernoulli distribution as the basis for our model. The probability density function is then defined as: *p*(*g*_1_ = *g*_2_|*M*, *H*) = 1 − *p*(*g*_1_ ≠ *g*_2_|*M*, *H*) = 1 − *q*. The unknown parameter, *q*, describes how likely a patient is to change gender or be entered incorrectly in the database. The prior, *p*(*q*|*H*, *M*) encodes our prior knowledge on *q* how likely a patient is to change gender or be entered incorrectly in the database. For our use case, we give *p*(*q*|*H*, *M*) a uniform distribution, running from 0.99 to 1.0. In order to calculate the Bayes factor in Eq 4, we need to integrate the probability densities over all a priori possible values of *q*. The numerator for Eq 4 therefore becomes:

$$\begin{array}{c} p(D|M,H)=\int p(D|q,H,M)p(q|H,M)dq \end{array}$$ (5)

The denominator of Eq 4 differs from the numerator as we are testing the alternatives hypothesis *K*, i.e. that *g*_1_ and *g*_2_ are not from the same patient. Our likelihood, *p*_*i*_(*g*_*i*_|*r*, *K*), therefore describes the probability of record *i* having *g*_*i*_ = male or female. Our parameter *r* parameterises the chance of picking at random a pair of people with different gender. In an even society, *r* = 0.5. To take into account slight variations in gender ratios, we allow *r* to vary between 0.4 and 0.6.

$$\begin{array}{c} p\left(D|M,K\right)=\prod_{i=1}^{2}\{\int p\left(r|K\right)\;p_{i}\left(g_{i}|r,K\right)\;dr\} \end{array}$$ (6)

We can now take every possible combination of patient records from year 1 and year 2, and calculate the Bayes factor for each combination. As there are only two possible outcomes (i.e. gender is the same or not), it follows that there are only two values of the Bayes factor.

### Bayes factor calculation for BMI

As an example of using continuous, time-varying, data, we will present steps of the Bayes factor calculation for BMI. Our model for BMI describes the probability of two records belonging to the same patient given the data, D, where *D* = {*BMI*_1_, *BMI*_2_} (the BMI values from the records from year 1 and year 2 respectively). Unlike gender, BMI is a function of age, and so we use the BMI curves from [16], and assume a patient follows a mean percentile *η*. The numerator of the Bayes factor (or marginalised likelihood for hypothesis *H*) is:

$$\begin{array}{c} p(D|M,H)=\int p(\eta |H)p(D|\eta ,H)\;d\;\eta \end{array}$$ (7)

Our prior on percentiles, *p*(*η*|*H*) is the normal distribution modelled on the [16] curves, while *p*(*D*|*η*, *H*) is the likelihood of measuring a BMI of *BMI*_*i*_ in year *i*, with error measurement *σ*_*i*_, given the patient lies on percentile *η*. We assume a Gaussian likelihood and so *p*(*D*|*η*, *H*) becomes:

$$\begin{array}{c} p\left(D|\eta ,H\right)=\frac{1}{{\left(2\pi \right)}^{1/2}\sigma }\exp\{-\sum_{i=1}^{L}\frac{{\left[BMI_{i}-f\left(\eta \right)\right]}^{2}}{2\sigma_{i}^{2}}\} \end{array}$$ (8)

Where *L* is number of years (2 in our case), and *f*(*η*) is governed by the BMI curves from [16], thereby taking into account the correlation between BMI from different years. In essence, we are fitting a BMI percentile to an individual patient and *p*(*D*|*η*, *H*) describes how good that fit is for a given *η*. For true matches, there will be a range of values *η* for which there is a high likelihood.

Our alternative hypothesis is that the BMI records are from different patients and is calculated by taking the product of the marginalised likelihood for each record.

$$\begin{array}{c} p\left(D|M,K\right)=\prod_{i=1}^{2}\{\int p\left(\eta_{i}|K\right)\;p_{i}\left(BMI_{i}|\eta_{i},K\right)\;d\;\eta_{i}\} \end{array}$$ (9)

The prior and likelihood are calculated in the same manner as the numerator, but with the important distinction that they are calculated individually for each record. In essence, this takes into account that you are more likely to find two random patients that lie near the 50th percentile than two patients that lie on the 5th percentile.

## Results

To validate the performance of the algorithm, we used the Danish data extracted from the ECFSPR, where records are linked from 2003-2009 using the manually created IDs. Table 1 shows the number of records per year and number of matches across each year. In total, there are 3066 records across the seven years and 2564 record matches.

**Table 1:** No of records in each annual snapshot of the Danish CF registry, as held by the ECFSPR. Number of matches correspond to links to records in the following year.

	2003	2004	2005	2006	2007	2008	2009
No. records	412	425	441	438	447	452	451
No. matches	407	421	431	430	434	441	

We used our algorithm to link patient records in each pair of adjacent years. Our algorithm provides a link between the pairs of records that match best and assigns a probability to each match. One can apply a *threshold* to this probability, discarding matches with probability below the threshold, and this threshold provides a tuneable parameter.

### Identifying errors in the Danish patient identifiers

**Fig 2:**
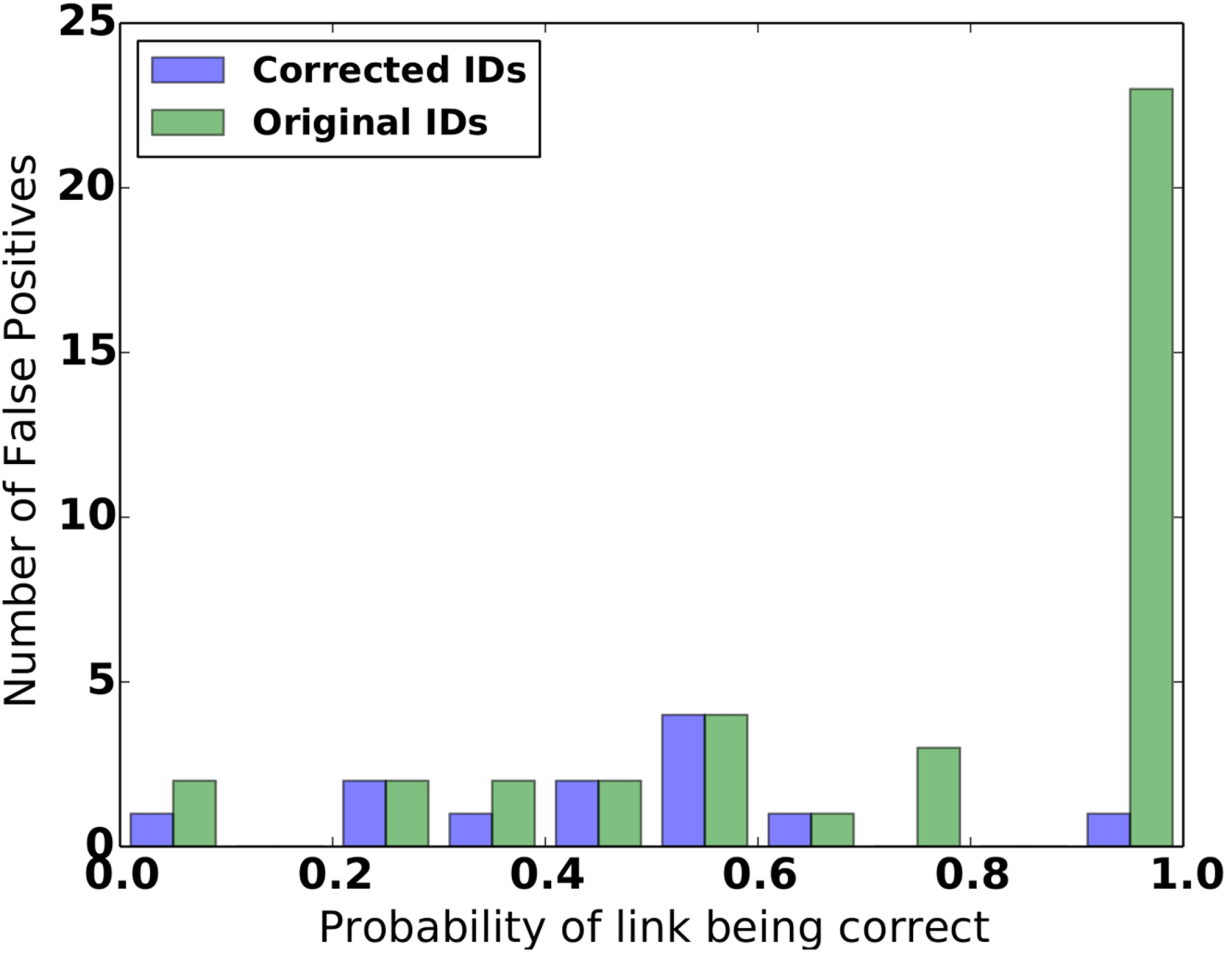
Histogram of the match probabilities for the false positives for first run of algorithm (in green) and final run with cleaned links (in blue). For the first run, our algorithm had assigned a ‘high probability of being a match’ to a significant number of false positives, the majority of which turned out to have incorrect IDs.

On first investigation, our algorithm found 2525 true positives (i.e. correct matches) and 39 false positives (i.e. matches that were not correct) for all probability thresholds. More worryingly, our algorithm had given the majority of false positives a high probability of being a match, as shown by the green bars in Fig 2.

A more detailed inspection of the wider data set for our false positives suggested that the matches seemed correct. We discussed these results with the Danish registry team who had provided the data. They confirmed that some of the identifiers were known to be wrong and provided the information necessary to correct these known errors.

On repeating our analysis the performance was much improved. However, there were still some false positives with a high probability. Further investigation by the Danish team (using additional personal identifiable information from the original Danish registry) showed that our algorithm had uncovered errors in the ECFSPR identifications that had not been previously known. These errors were then fixed.

### Final performance

On final evaluation, the number of true positives and false positives for all probability thresholds is 2552 and 12, while the ‘probability of link’ for the false positives, shown by the blue bars in Fig 2 is as we expect (e.g. there are very few with high probabilities). This compares well with the original ECFPSR links given to us, which achieve 2530 true positives and 26 false positives.

We assess the final performance of our algorithm using the standard precision and recall metrics. Precision can be defined, in terms of matches, as the number of correctly linked record pairs divided by the total number of linked record pairs (Eq 10). Recall is defined, in terms of matches, as the number of correctly linked record pairs divided by the total number of true match record pairs and is equivalent to sensitivity (Eq 11).

$$\begin{array}{c} Precision=\frac{\text{Number}\;\text{of}\;\text{true}\;\text{positives}}{\text{Number}\;\text{of}\;\text{true}\;\text{positives}\;+\;\text{Number}\;\text{of}\;\text{false}\;\text{positives}\;} \end{array}$$ (10)

$$\begin{array}{c} Recall=\frac{\text{Number}\;\text{of}\;\text{true}\;\text{positives}}{\text{Number}\;\text{of}\;\text{true}\;\text{positives}\;+\;\text{Number}\;\text{of}\;\text{false}\;\text{negatives}\;} \end{array}$$ (11)

**Fig 3:**
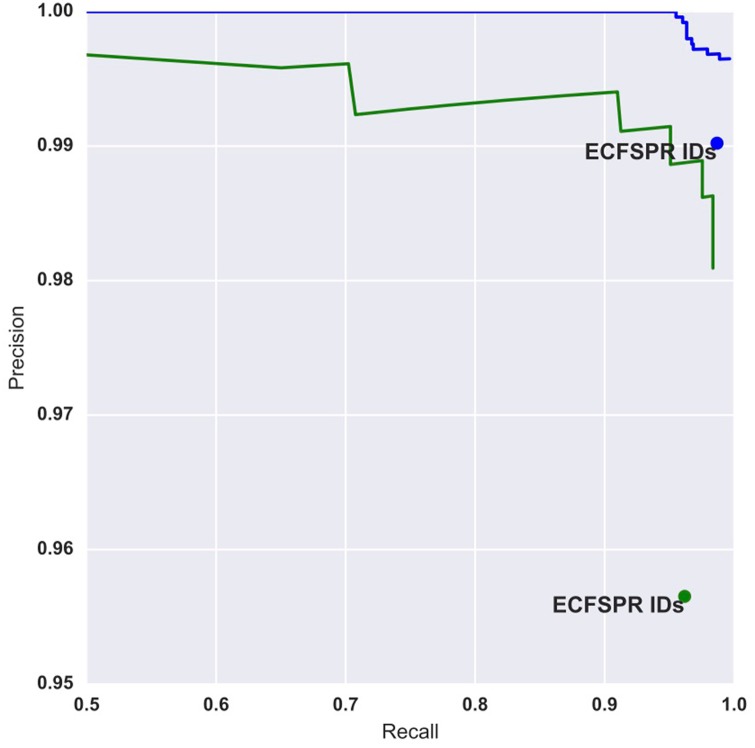
Precision-recall curves for our algorithm. The blue lines show the curve having applied our algorithm to consecutive years, with the blue marker representing the precision and recall obtained by the original ECFSPR IDs. For the same recall, our algorithm achieved a higher precision than the original identifiers. The green curve and marker indicate the performance having applied the algorithm to link Danish data between years 2003 and 2009.

The original identifiers provided by the ECFSPR, give a precision and recall value of 0.990 and 0.987 respectively. For our algorithm, one can use the probability threshold at which matches are accepted to tune either precision or recall at the expense of each other. For example, if one requires the algorithm to detect all of the true matches at the expense of picking up a few more mismatches, then a low probability threshold can be chosen. If however, it is important that all matches found by the algorithm are correct, at the expense of missing a few then one can choose a high probability threshold. Fig 3 shows the precision recall curve for our algorithm, alongside the precision and recall values from the original identifiers provided by the ECFSPR. For the same recall obtained by the original identifiers, our algorithm achieves a precision of 0.997.

By looking at the Bayes factors, we are able to get an indication of what variables are providing the most information in the linkage process. As expected, gender provides very little information, age at diagnosis provides some additional information, where as BMI and height provide a lot of certainty in the linkage. Genotype is an interesting variable in that the information content is very variable. For the most common CF genotype, df508 homozygous, genotype provides little information for linking, where as a rarer CF genotype will provide information on a similar scale to BMI. As countries have different ratios of genotypes, the amount of information provided by genotype will change across country.

### Linking across a 6 year span

One of the advantages of using our Bayesian model framework is that it allows us to incorporate prior knowledge and factors that vary with time. The way that variables such as BMI track with age, can be taken as prior knowledge for example from the BMI curves in [16]. By incorporating this knowledge into our probabilistic framework we can still link records even when separated by a number of years.

As a proof of concept, we have reapplied our algorithm to link the Danish ECFSPR records from 2003 directly with records in 2009 (i.e. rather than through sequential linkage of each year). The green lines in Fig 3 shows the precision recall curve. The original ECFSPR identifiers achieve a precision and recall of 0.957 and 0.962 respectively, as shown by the green dot. For the same recall value, our algorithm achieves a precision of 0.986 and although not as impressive as our overall performance for linking consecutive years, it still out performs the original identifiers.

## Discussion

The potential value of longitudinal studies is undisputed. However, when reviewing seven large international cohort studies on early human development from the integrated epidemiology unit in Bristol University, Richmond and co-authors found the outcomes are often not reproducible making causal inference problematic [28]. Difficulty in reproduction may stem from systematic errors in the ways the studies were conducted or their data were recorded.

Similar issues of data reliability can occur with retrospective registry data and broadly, there are two approaches to reduce error rates. First, it may be necessary to construct larger samples and then subdivide to measure systematic differences against a set standard and second, it is desirable to conduct independent audit of the validity of the data, preferably as close to the point of data entry as possible. In the work reported here, we can undertake both tasks. We show not only that new longitudinal data sets can be constructed at a patient level in the absence of unique identifiers but also that the validity of any existing and supposedly ‘gold standard’ identifiers can also be verified. In the proof of principle ‘test case’ reported here from the Danish sub-set of the ECFS data, the algorithm flagged up 27 patients whose data in the current ECFSPR registry was incorrect. In this capacity, the algorithm has been used to significantly clean up data. It has also been demonstrated that the algorithm can create very high quality identifiers, achieving a precision value of 0.997 which is higher than the original IDs for the same recall value of 0.987.

In the current standard of data in the cross-sectional ECFPR, both of these issues are important. It is known there are errors in the ECFSPR identifiers (e.g. significant errors in the ECFSPR Danish identifiers have been identified in this work) and about a third of the registry have intermittent data without consistency of identifiers [29]. This algorithm could be used to address both issues. It is likely that these issues will be faced by nearly all clinical registries aiming to construct longitudinal data. E.g. even in databases with minimal human data entry and linkage established through precise identifiers such as name and postcode, errors will arise though patient relocation. So this technique could have wide applications. Though performance and precise method of application will depend on the available factors, the nature of the patients and the size of the database.

The limitations of this study are that only one subset of data has been explored within one given data repository, so while the method is generalisable, the input parameters would need to be tailored to each specific case and the sensitivity and specificity apply only to this data set. It is also a limitation that the original data set could not be used as a gold standard and the true linkages had to be reassessed after the analysis, so while careful to correct the test information without bias, this study is not a truly blind assessment of the method. The analysis has also only been applied to each pair of years but future work could extend this to consider all years simultaneously. We also note that as treatments and approaches to CF change, the value of parameters used for linking may vary. A good example is the introduction of neonatal screening programs, which will drastically reduce the variation in age at diagnosis and in turn rendering it less informative for linking. To counteract this, other variables may become more appropriate and could be included into our framework as alternatives, such as weight at birth.

The joining of cross sectional datasets using the method is not without its ethical problems. ECFSPR and others take robust steps to protect individual identifiers though the use of trusted third parties and aggregation of rare genotypes that might identify a unique person by accident (www.ecfs.eu/registry). Yet, it has long been recognised that data held within registries are best described as semi-anonymous or quasi anonymous or pseudonymous because through a combination of factors it may be possible to identify an individual. Indeed the Bayesian approach does explicitly combine factors to assess the probability that a given dataset belongs to a given individual. However, it should be stressed that this identification is only internal to the database. Identifying that two records are highly likely to belong to the same patient but that does not mean one can identify the patient in the world, not least because there are many more people in the world than there are in the database. It is also unlikely that the act of linking records makes the data significantly less anonymous. If the data factors collated year-on-year are the same then linkage does not increase the number of factors. The value-added by the linkage is in the trends of these factors which adds little to the identification (e.g. if you know age, gender, height, weight, BMI etc, then the rate of change in these parameters doesn’t add much to your ability to identify the individual).

Nevertheless, this causes practical problems for certain countries because the consent might explicitly forbid or more commonly not directly permit the joining of individual datasets in order to analyse the data (despite its utility for the patient and the understanding of the natural history or are complications of a therapy). There is a clear unmet need for longitudinal analysis of data and the work reported here provides proof of principle that a set of bespoke tools can be custom built for a given registry. Before this can happen, consent needs to be modified to explicitly point out to those donating their data, that use of that data to create longitudinal datasets is absolutely vital to assess the new and exciting impacts of disease modifying therapies that are on the horizon [30, 31].

To summarise, this paper has shown how adopting a Bayesian model framework used for probabilistically linking records in astronomy, can be successfully applied to European Cystic Fibrosis Society Patient Registry data. This method can create longitudinal samples where none existed *and* validate pre-existing patient identifiers. In this specific case of the Danish registry data, often considered as the gold standard, this automated approach is *better* than the existing identifiers.

The methods in this paper has a number of additional implications for the efficiency of data handling within registries. They could be used to minimise duplication of patients for example by tracking which patients move between centres (students are a good example), functions that are additional to correcting identifiers as our incidental findings showed herein. For research, the approach may be of value in identifying such as identifying matched controls (with nearly identical characteristics save one parameter such as say BMI or lung function) or patients who vector differently and can be grouped by disease severity.

Our prototype code, along with an example Jupyter notebook to both generate mock data and apply algorithm, are made available at https://github.com/pdh21/problink.
